# Overwhelming Sepsis due to Capnocytophaga canimorsus in an Immunocompetent Individual: A Rare Case Study

**DOI:** 10.7759/cureus.10177

**Published:** 2020-09-01

**Authors:** Vinay Edlukudige Keshava, Harsh V Bhavsar, Nicholas Ghionni, Riaz H Baba, William Mcnamee

**Affiliations:** 1 Internal Medicine, Mercy Catholic Medical Center, Darby, USA; 2 Internal Medicine, Mercy Catholic Medical Center, Philadelphia, USA; 3 Internal Medicine - Infectious Disease, Mercy Catholic Medical Center, Darby, USA

**Keywords:** sepsis, thrombotic thrombocytopenic purpura, shewanella putrefaciens, capnocytophaga canimorsus

## Abstract

We report this rare case of fatal fulminant sepsis in a 42-year-old African American female who presented with a three-day history of generalized pain and an evolving rash all over her body. On presentation, the patient was tachycardic, borderline hypotensive, and febrile. Physical examination was significant for diffuse petechiae and ecchymoses over the extremities, torso, and the face, especially confluent over her thighs and lower abdomen. She was admitted to the ICU, and initial investigations revealed a normal leukocyte count and hemoglobin but severe thrombocytopenia, elevated creatinine, blood urea nitrogen (BUN), bilirubin, transaminases, and an elevated INR. She also had a high anion gap metabolic acidosis with elevated lactate. Chest and abdomen CT findings were nonspecific, demonstrating fluid surrounding both kidneys, a moderate amount of fluid in the pelvis, and alveolar opacities at the bases of both lungs. Initial working diagnoses were a septic shock, thrombotic thrombocytopenic purpura (TTP), and vasculitis. She was initiated on broad-spectrum antibiotic coverage with vancomycin, piperacillin/tazobactam, and doxycycline pending culture reports. After a few hours, she became progressively hypothermic, developed disseminated intravascular coagulation (DIC) and hemodynamic instability, and was intubated due to acute hypoxic and hypercapnic respiratory failure. She progressively worsened hemodynamically with multi-organ dysfunction, and ultimately was pronounced dead roughly 18 hours after initial presentation. Blood cultures grew a Gram-negative organism, initially reported as *Shewanella putrefaciens*, but subsequently confirmed as *Capnocytophaga canimorsus*.

## Introduction

*Capnocytophaga canimorsus* is a fastidious Gram-negative organism that is a part of the gingival flora of cats and dogs, which can cause life-threatening infections in humans. It is the most common pathogen implicated in dog bite infections. Clinical presentation ranges from localized soft-tissue infections around the bite to fulminant fatal septicemia [1]. Early diagnosis and initiation of appropriate treatment remain the mainstay in the management of this lethal infection but are limited by the lack of modalities to accomplish the same. Diagnosis is even more challenging and complicated by a wide range of varied symptoms and signs, with a list of numerous differential diagnoses. Here, we present a case of overwhelming sepsis in an immunocompetent individual, caused by *C. canimorsus*. We also attempt to address the challenge in the diagnosis of this organism and review the current literature.

## Case presentation

Mrs. S.C. is a 42-year-old African American female who presented with a three-day history of progressively worsening generalized body aches and an evolving rash all over her body. Past medical history was only significant for intermittent asthma, for which she was on an albuterol inhaler as needed. She also admitted to taking a five-day course of azithromycin recently for an upper respiratory tract infection, and ephedrine, prescribed by a local "weight-loss doctor." She denied any pet exposure/bites, reported being an avid hiker but had not hiked in the last two to three months. On presentation, she was febrile, hypotensive (blood pressure, BP 97/58 mmHg), tachycardic (106 beats per minute), and tachypneic (26 breaths per minute). Oxygen saturation was 93% on room air. Physical examination was significant for multiple diffuse petechiae and a few ecchymoses over bilateral upper limbs, abdomen, and back. The lesions had a reticular pattern of distribution over the lower limbs, more confluent in the thighs. Abdominal examination revealed diffuse tenderness to palpation, but with no evidence of rebound tenderness or organomegaly. Respiratory, cardiac, and neurological examinations were unremarkable.

**Figure 1:**
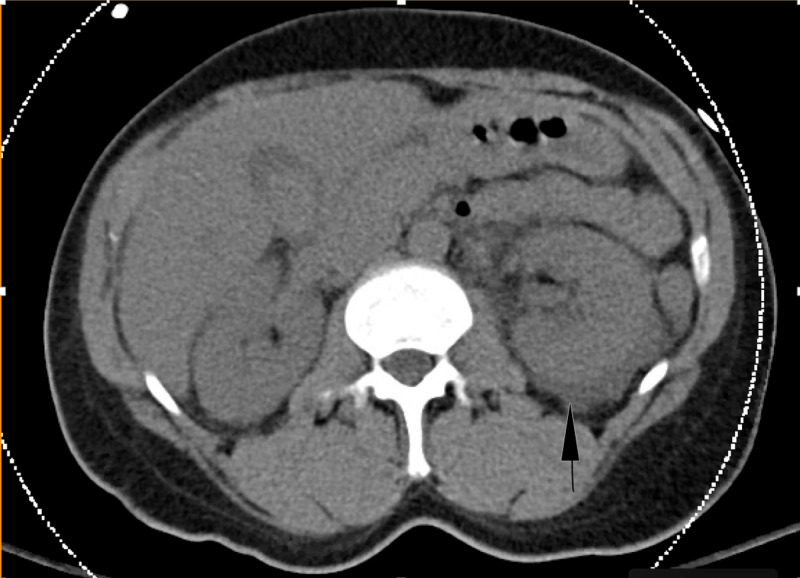
CT abdomen and pelvis without contrast: showing fluid surrounding both the kidneys with moderate amounts of fluid in the pelvis. Gall bladder showed a filling defect, thought to be secondary to sludge/calculus.

Initially laboratory investigations revealed a hemoglobin (Hb) of 13.3 g/dL (normal range=12-16), white blood cell (WBC) count of 4.5 x 109/L (normal range=4.5-11), platelet count of 17 x 109/L (normal range=150-450), sodium of 132 mEq/L (normal range=136-145), potassium of 5.1 mEq/L (normal range=3.5-5.0), chloride of 97 mEq/L (normal range=98-110), bicarbonate of 15 mEq/L (normal range=23-29), creatinine of 3.1 mg/dL (normal range=0.6-1.3), blood urea nitrogen (BUN) of 37 mg/dL (normal range=7-25), total bilirubin of 2.8 mg/dL (normal range=0.2-1.2), aspartate aminotransferase (AST) of 254 U/L (normal range=10-40), alanine aminotransferase (ALT) of 200 U/L (normal range=7-52), alkaline phosphatase (ALP) of 97 U/L (normal range= 35-110), C-reactive protein (CRP) of 312 mg/dL (normal range=<1.0), serum creatinine kinase of 595 U/L (normal range=26-140), lactate dehydrogenase of 282 U/L (normal range=140-280), INR of 3.0 (normal range=0.8-1.2), troponin I of 0.08 ng/mL (normal range=<0.05). Urinalysis was positive for large occult blood with 6-10 red blood cells/high power field, a specific gravity of 1.030, and a pH of 5.0. Venous blood gas was obtained, which showed a pCO2 of 68.2 mmHg, bicarbonate of 14.8 mmol/L, and lactic acid of 6.3 mmol/L showing a significant, high anion gap metabolic acidosis. A CT scan of the abdomen showed nonspecific findings like fluid surrounding both the kidneys with moderate amounts of fluid in the pelvis (Figure 1). Gall bladder showed a filling defect, thought to be secondary to sludge/calculus, but other intra-abdominal organs, including the spleen, were visualized to be within normal limits.

While still being evaluated in the ED, she had supraventricular tachycardia with a drop in her systolic BP to 92 mmHg. Per ACLS protocol, she got synchronized cardioversion for two cycles, and an amiodarone drip was initiated following a bolus, controlling her arrhythmia. She was subsequently admitted to the ICU for further close monitoring. The broad differential diagnoses included septic shock, thrombotic thrombocytopenic purpura (TTP), and vasculitis. She was started on broad-spectrum antibiotic therapy with vancomycin, piperacillin/tazobactam, and doxycycline at renally adjusted dosages. Peripheral smear performed initially did not show evidence of hemolysis. The auto-immune work-up revealed negative results for antinuclear antibody (ANA), perinuclear anti-neutrophil cytoplasmic antibody (p-ANCA), and cytoplasmic ANCA (c-ANCA); C3 complement was slightly decreased at 73 mg/dL (normal 90-180).

Over the next few hours, she got progressively hypotensive, and vasopressor agents like norepinephrine and vasopressin had to be initiated for optimization of her hemodynamics. She continued to deteriorate rapidly, with progressively worsening altered mental status clinically. Her cardiac monitor demonstrated pulseless electrical activity, which necessitated the initiation of ACLS protocol. She was intubated, mechanically ventilated, and return of spontaneous circulation was achieved after three rounds of cardio-pulmonary resuscitation. Her repeat laboratory studies showed an INR of 6.5, AST of 1136 U/L, ALT of 1049 U/L, and lactic acid of 20.0 mmol/L, indicating worsening multi-organ dysfunction syndrome (MODS) and disseminated intravascular coagulation (DIC). Eventually, despite continued resuscitation efforts and hemodynamic support, her clinical status continued to deteriorate, and she was pronounced dead 18 hours after her arrival at the hospital. Post-mortem diagnosis per the pathologist was noted as TTP.

However, after two to three days, the blood culture showed growth of Gram-negative bacilli in the anerobic bottles, which was identified initially as *Shewanella putrefaciens*. Given the rarity of Shewanella species causing overwhelming systemic sepsis syndrome, the sample was sent for further testing for confirmation. After 14-15 days, the Gram-negative organism was finally identified as *C. canimorsus*.

## Discussion

*Capnocytophaga canimorsus*, formerly known as CDC group DF-2 (dysgonic fermenter 2), is a Gram-negative, fastidious organism, known to be *Capnophilic bacillus* (growth is enriched in a medium with CO2). It differs from other members of the Capnocytophaga family, like *C. gingivalis*, *C. sputigena*, and *C. ochracea* (DF-1 organisms) in testing oxidase and catalase-positive, but resembles the members of the family in being fermentative and exhibiting gliding motility [1]. It is a part of the normal gingival flora of cats and dogs (canimorsus is Latin for "dog bite") but is implicated in many infectious syndromes in humans, mainly septicemia, meningitis, endocarditis, and ocular infections [1].

Although rare, many case reports of *C. canimorsus* infections have been reported worldwide, with an age range of 4 months to 77 years. Men are more commonly infected (74%) than women. Although infections have known to occur in individuals with no pre-existing long-standing medical conditions (like our patient), due to its slow growth and low virulence, it more commonly affects patients with risk factors such as immunosuppression, asplenia, alcoholism, hematological malignancies, and cirrhosis [2-3].

Approximately 54% of the infections with *C. canimorsus* are due to an animal bite, but Lion et al. reported a shockingly high infection rate of 27%, just by mere exposure to animals [4]. Furthermore, 8.5% of the infections are caused by scratches. Commonly, it takes two to three days after contact with the animal for the infection to develop, but on some occasions, it has been shown to present as late as four weeks following the exposure. Although our patient refused any animal bites/pet animal exposure, she could have forgotten about exposure in the past week or two, more so if it were trivial.

Interestingly, our patient's working diagnosis also included TTP initially, as she met four criteria among the classical pentad (fever, thrombocytopenia, renal failure, and neurological involvement). The autopsy report also concluded TTP to be the most likely cause of death, but this had to be excluded later, given the positive blood cultures. Brichacek et al. also reported a similar case [5] where the clinical presentation was thought to be an atypical TTP secondary to *C. canimorsus* sepsis. It is worthwhile to mention that bacterial cytotoxins have been implicated in the causation of TTP, especially agents like *Escherichia coli*, *Shigella dysenteriae*, and *Salmonella typhi*.

Accurate diagnosis without delay and initiation of appropriate treatment is of paramount importance for patient survival, given the high mortality rate associated with *C. canimorsus* infection. It is often very challenging to detect and isolate it. In our case, utilization of the VITEK-2 system (bioMérieux, Marcy-l'Étoile, France) provided an incorrect result initially, identifying the organism as *S. putrefaciens* (a Gram-negative, pleomorphic marine organism which has rarely been reported to cause human infection). After this, the specimen was sent out to an out of hospital specialized laboratory for definitive confirmation. The VITEK-2, used by many hospital systems across the country, is a second-generation rapid automated system, originating from the VITEK system family which was created in the 1970s for identification and antibiotic susceptibility testing after preparation of initial inoculum, along with phenotypic and MIC (minimum inhibitory concentration) testing for identification [6]. It is a critical aspect of microbiological testing with the goals of testing to identify possible drug resistance in common pathogens and investigate drug susceptibility, with the most widely used instruments using broth micro-dilution or rapid automated instrument methods, which are currently broadly available in the market [7].

The patient referenced in this case report had rapid clinical deterioration. Given the rapidity with which she progressed, it is unclear if rapid identification of the correct microbiological organism could have indirectly affected the treatment, therapy options, and prognosis. Janda et al. [8] discussed the inability of many laboratories to identify *C. canimorsus* isolates with reasons of mislabeling cited as lack of commercial systems for identifying fastidious organisms and lack of familiarity with the organism. Interestingly, the same article asserts after study of laboratory results over three decades, that the low correct identification rate (32%) suggests that the actual rate of *C. canimorsus* infections in the general population may be higher than estimated, particularly if samples are not sent to laboratories equipped the ability to provide definitive identification [8]. There are many molecular approaches to identification that could help prevent incorrect identification, two of which are 16S rRNA gene sequencing or polymerase chain reaction (PCR) assays, the results of this case also took several weeks to return.

## Conclusions

This case highlights the clinical presentation, potential risk factors, differential diagnoses, and the obstacles in definitive and early diagnosis, which need to be considered with *C. canimorsus* infections. It also highlights the importance of history suggestive of a potential animal exposure which could clinch the diagnosis early, in an appropriate clinical setting. We also opine that newer, cheaper, rapid, and broadly available methods for identification and diagnosis are needed to quickly provide appropriate and effective care in zoonotic infections such as *C. canimorsus*, whose case-fatality rates remain high.
